# *Chlamydia trachomatis*, Human Immunodeficiency Virus (HIV) Distribution and Sexual Behaviors across Gender and Age Group in an African Setting

**DOI:** 10.1371/journal.pone.0090174

**Published:** 2014-03-03

**Authors:** Joel Fleury Djoba Siawaya

**Affiliations:** Unité de Recherche et de Diagnostic Spécialisé, Laboratoire National de Santé Publique (LNSP) BP 10736, Libreville, Gabon; Auburn University, United States of America

## Abstract

**Objective:**

The purpose of this study was to (1) describe the distribution of *Chlamydia trachomatis* (CT) and Human Immunodeficiency Virus (HIV) cases across gender and age groups in Libreville (Gabon); (2) examine Gabonese Sexually Transmitted Infections (STIs)-related risk behaviour.

**Methods:**

The sampled population was people attending the *“Laboratoire National de Santé Plublique”*. Between 2007 and 2011, 14 667 and 9 542 people respectively, were tested for CT and HIV infections. 1 854 of them were tested for both infections. We calculated CT and HIV rates across gender and age groups. Also analysed was the groups' contribution to the general CT and HIV epidemiology.

STIs-related risk behaviours were assessed in 224 men and 795 women (between July 2011 and March 2013) who agreed and answered a questionnaire including questions on their marital status, number of sex partners, sexual practices, history of STIs, sex frequency and condom use.

**Results:**

Data showed a 24% dropped in the CT infection rate between 2007 and 2010, followed by a 14% increase in 2011. The HIV infection rates for the same period were between 15% and 16%. The risk of a CT-positive subject getting HIV is about 0.71 times the risk of a CT-negative subject.

Young adult aged between 18 and 35 years old represented 65.2% of people who had STIs. 80% of women and 66% of men confessed to an inconsistent use of condoms. 11.6% of women and 48% of men declared having multiple sex partners. 61% of questioned women and 67% of men declared knowing their HIV status.

**Conclusions:**

In this Gabonese setting, the population-aged from18 to 35 years is the most affected by STIs. Other matters of concern are the inconsistent use of protection and sex with non-spousal or non-life partners.

## Introduction

*Chlamydia trachomatis* (CT) is the most common sexually transmitted infection (STI) worldwide with prevalence ranging from 1.5% to 5% in the developed world [1], [2], [3] and 3.7% to 15% in developing countries [4], [5], [6]. The human immunodeficiency virus (HIV) infection on the other hand is the highest killing sexually transmitted infection. Worldwide, more than 30 million of people are living with HIV. In 2010 the number of new HIV infections was estimated at 2.7 million [7], whereas the number of people dying from Acquired Immunodeficiency Syndrome (AIDS)-related causes was 1.8 million [7].

Women, young people and people from modest backgrounds are thought to be particularly vulnerable to STIs [7], [8], [9], [10], [11], [12]. In 2009 in the USA, persons aged from 15 to 29 years represented 21% of the population but accounted for 39% of all new HIV infections [13]. In 2011, the Centre for Disease Control and Prevention (CDC) in the USA showed that 13.1% of students had four or more sex partners [14].

According to the 2011 UNAIDS report 68% of people living with HIV resided in sub-Saharan Africa [7]. The report also revealed that the continent accounted for 70% of new HIV infection cases with 1.2 million deaths [7].

Controlling and reducing the transmission of STIs remain public health priorities in Africa where, high-risk behaviours, precariousness, low literacy rate and the lack of proper sexual education are in the list of STIs programs bottlenecks [8], [9], [10], [11], [15], [16]. In Uganda, between 2001 and 2005 there was a 5% (from 24% to 29%) increase in sex with multiple partners and sex with non spousal partners in the population of men whose age is between 15 and 49 years old [17].

Although few reports on behavioural interventions and STIs incidence exist [18], we are in agreement with the thought that reducing STIs-related risk behaviours among youth and other vulnerable population groups is essential for the effectiveness of any prevention programs [17], [19], [20], [21], [22], [23], [24]. However, optimization of developing countries' response to prevent the transmission of STIs requires both information and the capacity to use this information to build an efficient response. Understanding STIs epidemiological dynamics, population behaviours and what drives them is crucial [11], [22].

The HIV prevalence in Gabon at the time of this study was estimated around 5% [25]. The country has the particularity of having a high literacy rate (87%). The school enrolment rate of children aged less than 18 years old is estimated at 96.5%. No reported data exists regarding CT infection and no reliable data exists on both CT and HIV distribution across gender and age groups. CT being the most common sexually transmitted disease, analysing its rates and distribution in a population where HIV rate is relatively low can give a proper insight on people's safe sex practices, allow the identification of populations at risk and provide a good indicator on what could be the progression of HIV infection in the absence of proper interventions.

The present study represents a unique effort not only to obtain the distribution of CT and HIV infections across gender and age groups in the particularly interesting setting; but also to have a view of the evolution of the selected STIs and highlight the determinants of sexually transmitted infections control in a relatively educated African population. This is very important because as stated by *Bertozzi et al*., [22]: “*insufftcient data for intervention strategies leaves programs managers to operate in a fog of uncertainty*”.

## Materials and Methods

Voluntary testing for STIs is not common practice in Gabon. Therefore clinicians are encouraged to include HIV testing when prescribing blood exams to their patients. This practice allows many to know their STIs status.

This study was carried out at Gabonese National Laboratory of Public Health (LNSP). Data used in the study comes mainly from subjects who got tested for STIs after consulting a clinician. The Gabonese National Laboratory of Public Health review board has approved the present study. When required, all patients signed an informed consent form.

### Evolution of CT and HIV infection cases and distribution across gender and age groups between 2007 and 2011

Between January 2007 and December 2011, 14 667 and 9 542 people attending the LNSP were respectively tested for CT and HIV infections. 1 854 of them were tested for both infections. Serological testing was the method used to diagnose CT *trachomatis* (IgG/IgA) and HIV (Ab/Ag). The population age range was 13 to 85 years old.

### Association between CT and HIV infections

The association between CT and HIV infections (*relative risk and odds ratio* at a 95% confidence interval) was assessed using a contingency table. GraphPad Prism version 6 was the software used for the analysis.

### Assessment of STIs-related risk behaviours

Between July 2011 and March 2013, 224 men and 795 women who attended the LNSP for fertility and infectious diseases check-up agreed to participate in this study. After signing an informed consent form, all the subjects were given a questionnaire including questions on their marital status, number of sex partners, sexual practices, history of STIs, sex frequency and condom use. We compiled and analyzed data collected (using GraphPad Prism version 6 was the software).

## Results

### Evolution of CT and HIV infections (table 1)

**Table 1:** Chlamydia trachomatis (CT) and the human immunodeficiency virus (HIV) infections a the Gabonese National Laboratory of Public Health (2007-2011).

	2007	2008	2009	2010	2011
***Chlamydia***					
**Number of patients tested in the year**	[F: 1719 (77%); M: 510 (23%)]	3596 [F: 2887 (80%); M: 709 (20%)]	2574 [F: 2075 (80.6%); M: 299 (19.4%)]	3308 [F: 2641 (80%); M: 667 (20%)]	2960 [F: 2341 (79%); M: 619 (21%)]
**Seropositive**	1657 [74.3%] [F: 77.6%; M: 22.4%]	2546 [71%] [F: 79%; M: 21%]	1248 [48.5%] [F: 86%; M: 14%]	1554 [47%] [F: 85%; M: 15%]	1802 [61%] [F: 83%; M: 17%]
**Active Chlamydia Infection**	1485 [65.4%] [F: 75%; M: 25%]	2372 [66%] [F: 77%; M: 23%]	1123 [43.6%] [F: 80%; M: 20%]	1396 [42%] [F: 81%; M: 19%]	1620 [55%] [F: 88%; M: 12%]
**Seronegative**	572 [25,7%] [F: 74%; M: 26%]	1050 [29%] [F: 75.6%; M: 24.4%]	1326 [51.5%] [F: 76%; M: 24%]	1754 [53%] [F: 75.5%; M: 24.5%]	1158 [39%] [F: 73%; M: 27%]
***HIV***					
**Number of patients tested in the year**	73 [F: 59 (81%); M: 14 (19%)]	2671 [F: 2162 (81%); M: 509 19%]	2071 [F: 1677 (81%); M: 394 (19%)]	2578 [F: 1950 (75.6%); M: 628 (24.4%)]	2149 [F: 1600 (74.5%); M: 549 (25.5%)]
**Seropositive**	12 [16.4%] [F: 75%; M: 25%]	398 [15%] [F:75%; M: 25%]	285 [16%] [F: 82%; M: 18%]	386 [15%] [F: 75.4%; M: 24.6%]	323 [15%] [F: 73%; M: 27%]
**Seronegative**	61 [83.6%] [F: 82%; M: 18%]	2273 [85%] [F: 73.6%; M: 26.4%]	1786 [86%] [F: 81,6%; M: 18,4%]	2192 [85%] [F: 76%; M: 24%]	1826 [85%] [F: 75%; M: 25%]
***Chlamydia/HIV***					
**Number of patients tested for both infection in the year**	20 [F: 14 (70%); M: 6 (30%)]	472 [F: 371 (78.6%); M: 101 (21.4%)]	465 [F: 292 (63%); M: 173 (37%)]	478 [F: 357 (75%); M: 121 (25%)]	422 [F: 328 (78%); M: 94 (22%)]
**Chlamydia (−)/HIV (−)**	7 [35%] [F: 57%; M: 43%]	141 [30%] [F: 72%; M: 28%]	182 [39%] [F: 77%; M: 23%]	266 [55.6%] [F: 74%; M: 26%]	167 [40%] [F: 75%; M: 25%]
**Chlamydia (+)/HIV (+)**	2 [10%] [F: 100%; M: 0%]	31 [6.5%] [F: 84%; M: 16%]	10 [2%] [F: 80%; M: 20%]	15 [3%] [F: 87%; M: 13%]	25 [6%] [F: 80%; M: 20%]
**Chlamydia (+)/HIV (−)**	10 [50%] [F: 80%; M: 20%]	272 [57.5%] [F:81%; M: 19%]	249 [54%] [F: 50%; M: 50%]	163 [34.4%] [F:76%; M: 24%]	217 [51,4%] [F: 80%; M: 20%]
**Chlamydia (−)/HIV (+)**	1 [5%] [F: 100%; M: 0%]	28 [6%] [F: 82%; M: 18%]	23 [5%] [F: 82.6%; M: 17.4%]	34 [7%] [F: 70.6%; M:19.4%]	11 [2.6%] [F: 64%; M: 36%]

The rate of people with positive CT serology dropped from 74% in 2007 to 47% in 2010. In 2011 this rate increased reaching 61%. Active CT infection rate followed the same trend, going from 65.4% in 2007 to 42% in 2010. In 2011 the rate of active CT infection went back up to 55%. HIV rate among people tested at the LNSP stayed relatively constant, between 15% and 16.4%.

### CT and HIV Co infection

Data shows that between 2007 and 2011, the CT-HIV Co infection rate followed the CT trend, going respectively from 10% (2007), 7% (2008), 2% (2009), 3% (2010) and 6% (2011). Between 2007 and 2011, the prevalence of CT infections among HIV-positive people was 46% (range: 30.6%–69.4%). In 2011 69.4% of HIV infected people had CT.

### Association between CT and HIV infections

The odds ratio of getting HIV with positive CT serology in our setting was 0.71 (95% confidence interval (CI): 0.53 to 0.97). The relative risk of getting HIV when infected with CT was 0.74 (95% CI: 0.56 to 0.98). The risk of a CT-positive subject getting HIV is about 0.71 times the risk of a CT-negative subject. Details on CT and HIV-infection prevalence for the period going from 2007 to 2011 are confined in table 2.

**Table 2:** Chlamydia trachomatis (CT) and human immunodeficiency virus (VIH) infection prevalence for the period 2007–2011.

	2007		2008		2009		2010		2011	
	Chlamydia	HIV	Chlamydia	HIV	Chlamydia	HIV	Chlamydia	HIV	Chlamydia	HIV
**All subjects**	1657	12	2546	398	1248	285	1554	388	1809	323
**Under 18 years old**	101 (6.1%)	0 (0%)	138 (5.4%)	20 (5%)	113 (9%)	15 (5.3%)	35 (2%)	2 (0.5%)	23 (1.3%)	11 (3.4%)
**18–35 year old**	1164 (70.2%)	7 (58%)	1910 (75%)	238 (60%)	866 (69.5%)	172 (60.4%)	1025 (66%)	211 (54.4%)	1099 (60.7%)	149 (46.1%)
**Over 35 year old**	392 (23.7%)	5 (42%)	498 (19.6%)	140 (35%)	269 (21.5%)	98 (34.4%)	494 (32%)	175 (45.1%)	667 (37%)	163 (50.5%)
**Pupils or Students**	450 (27.1%)	0 (0%)	1224 (48%)	95 (24%)	404 (32.4%)	35 (12.3%)	504 (32.4%)	36 (9.3%)	391 (21.6%)	21 (6.5%)

### CT and HIV infections cases distribution across gender and age groups (table 3)

**Table 3:** Chlamydia trachomatis (CT) and the human immunodeficiency virus (VIH) infections distribution across gender and age groups.

Patients tested for both infection between 2007 and 2011				
	HIV (+)	HIV (−)	Total	HIV-prevalence rate
**Chlamydia (+)**	83	911	994	8.3%
**Chlamydia (−)**	97	763	860	11.3%
**Total**	180	1674	1854	9.7%
**CT- prevalence**	46.1%	54.4%	53.6%	

#### Population aged under18 years

The population aged less than 18 years old accounted respectively for 6.1% and 5.4% of people infected with CT for the years 2007 and 2008. After reaching a pic of 9% in 2009, this population only represented 1.3% of people infected with CT in 2011. In 2007 we had no record of HIV infection for the population aged less than 18 years old. In 2008, 2009 and 2011 respectively, 5%, 5.3% and 3.4% of people infected with HIV were under 18 years old.

#### Population aged 18–35 years

People aged 18 to 35 years old represented 70.2% of people infected with CT in 2007. This rate dropped to 60.7% in 2011. The same population represented 58% of people infected with HIV in 2007. This rate dropped to 46.1%, in 2011.

#### Population aged over 35 years

Between 2007 and 2011 the part of the population over 35 years old in the CT burden increased from 23.7% to 37%. This population accounted for 42% of people infected with HIV in 2007. This rate increased to 50.5% in 2011.

#### Students (High school and University)

Students' age range was 13 to 37 years old. They represented 20%, 48%, 32.4%, 32.4% and 21.6% of people infected with CT for the years 2007, 2008, 2009, 2010 and 2011 respectively. They accounted for 24% of people infected with HIV in 2008. This rate dropped to 6.5% in 2011.

### STIs-related risk behaviours

**Figure 1:**
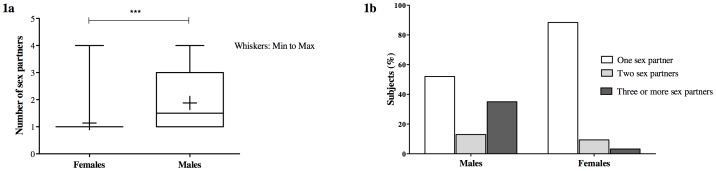
Sex with multiple partners. (a) Number of sex partners by gender groups: men had in average 1.9 sex partners and women 1.1. The difference between the groups was statistically significant (p = 0.0001; [Mann Whitney test]). (b) Percentage of men and women with one, two and three or more sex partners: a higher rate of men engaged in sex with multiple partners compared than women.

**Figure 2:**
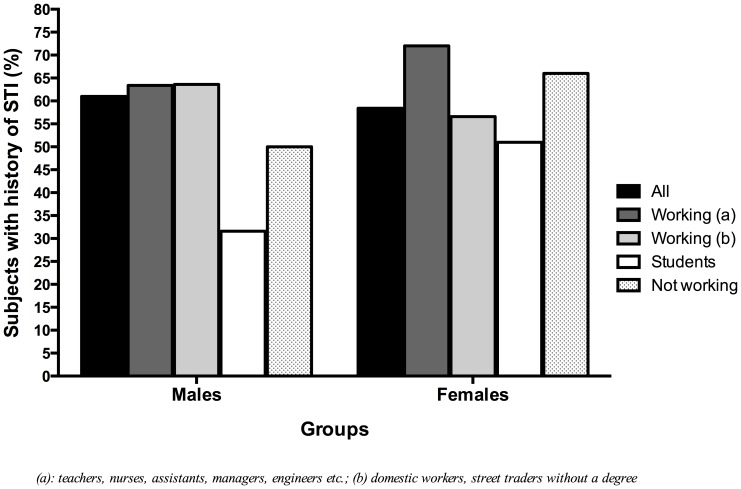
History of STIs in men and women. Overall the percentages of male and female with a history of STIs were similar. Within the male group, student and unemployed male were less affected by STIs (31.6% and 50% respectively). Among females student and less educated employed women were less affected by STIs (51% and 56.6% respectively).

The frequency of sexual intercourse was higher in men compared to women. On average men had sex 10 times per month and women four times per month. Also men had significantly more sexual partners than women (p = 0.0001[Mann Whitney test]) (figure 1a and 1b). No real differences were seen between men and women when comparing their STIs history. 61% of men and 58.4% of women had a history of STIs (figure 2).

#### STIs-related risk behaviours in men

225 sexually active men from different backgrounds aged between 19 and 61 years old were included in this study. Of these men 48% declared having multiple sexual partners. 46.3% of men engaged in a committed relationship had sex with non-spousal or non-life partners. 65.5% of men (51.2% of single men) admitted to an inconsistent use of condoms. 32% declared practicing cunnilingus (oral sex). 67% of men declared knowing their HIV status, whereas 33% declared not knowing their HIV status. Multiple sex partners were the highest in male students (59%) followed by educated workingmen (53%). More details on age and social groups behaviours are confined in table 4.

**Table 4:** Sexually transmitted infections -related risk behaviours in men.

N = 224				
Marital Status	**Committed Relationship (CR)**		**Single (S)**	
	***Engaged***	***Married***		
	49.3%	29.3%	21.4%	
*Use of condoms*	[A: 2%; O: 32%; ST: 30%; N: 36%]	[A: 0%; O: 21%; ST: 21%; N: 58%]	[A: 4%; O: 47%; ST: 32.6%; N: 16.4%]	
*2 or more sexual partners*	54%	38%	60%	
Sex Frequency	**More than twelve times a month**	**At least eight times a month**	**Less than eight times a month**	
	53.5%	25.5%	21%	
Social status	**Unemployed**	**Student**	**Employed (a)**	**Employed (b)**
	6 (2.7%)	22 (9.9%)	111 (49.8%)	84 (37.6%)
*Use of condoms*	[A: 0%; O: 33%; ST: 17%; N: 50%]	[A: 9%; O: 41%; ST: 32%; N: 18%]	[A: 2%; O: 35%; ST: 31%; N: 32%]	[A: 0%; O: 25%; ST: %; N: 51%]
*2 or more sexual partners*	33%	59%	53%	39%
Age groups	**18–35**	**Over 35**		
	122 [CR: 64%; S: 36%]	102 [CR: %; S: %]		
*Use of condoms*	[A: 3%; O: 33%; ST: 30.4%; N: 33.6%]	[A: 1.3%; O: 31%; ST: 24%; N: 43.7%]		
*2 or more sexual partners*	49%	46%		

(a): teachers, nurses, assistants, managers, engineers etc.; (b) domestic workers, street traders without a degree etc.; CR: committed relationship, S: single; A: always; O: often; ST: sometime; N: never, (*) more than 3 sexual partners.

#### STIs-related risk behaviours in women

795 sexually active women from different backgrounds aged between 14 years old and 55 years old were included in this study (table 5). 11.6% declared having more than one sexual partner. 8.5% of women engaged in a committed relationship had sex with non-spousal or non-life partners. 78% of single women declared the inconsistent use of protection when engaged in sexual activities. 61% of the questioned women declared knowing their HIV status, whereas 39% declared not knowing their HIV status. 58.4% of women had a history of STIs. 64% of women declared practicing cunnilingus (oral sex); multiple sex partners were the highest in educated workingwomen (14.3%) followed by female students (13%) (table 5).

**Table 5:** Sexually transmitted infections-related risk behaviours in women.

N = 795				
Marital Status	**Committed Relationship (CR)**		**Single (S)**	
	***Engaged***	***Married***		
	36%	10%	54%	
*Use of condoms*	[A: 2%; O: 13%; ST: 51%; N: 34%]	[A: 4.3%; O: 24%; ST: 39.1%; N: 32.6%]	[A: 0.5%; O: 21.3%; ST: 45.2%; N: 33%]	
*2 or more sexual partners*	11%	7.4%	14%	
Sex Frequency	**More than four times a month**	**At least four times a month**	**Less than four times a month**	
	33%	26.7%	40.3%	
Social status	**Unemployed**	**Student**	**Employed (a)**	**Employed (b)**
	173 (22.2%)	392 (50.3%)	132 (17%)	82 (10.5%)
*Use of condoms*	[A: 0%; O: 11.7%; ST: 52.6%; N: 35.7%]	[A: 2.4%; O: 23.2%; ST: 52.2%; N: 22.2%]	[A: 1.6%; O: 16%; ST: 39%; N: 43.4%]	[A: 0%; O: 17.7%; ST: 25.3%; N: 57%]
*2 or more sexual partners*	11%	13%	14.3%	5%
Age groups	**13–17**	**18–35**	**Over 35**	
	23 [CR: 8.7%; S: 81.3%]	653 [CR: 56.7%; S: 43.3%]	119 [CR: 65.7%; S: 34.3%]	
*Use of condoms*	[A: 0%; O: 26%; ST: 52%; N: 22%]	[A: 1.4%; O: 20%; ST: 48%; N: 30.6%]	[A: 1.7%; O: 11.5%; ST: 36.7%; N: 50.4%]	
*2 or more sexual partners*	22%	11.6%	10.4%	

(a): teachers, nurses, assistants, managers, engineers etc.; (b) domestic workers, street traders etc.; CR: committed relationship, S: single; A: always; O: often; ST: sometime; N: never.

## Discussion

The Gabonese National Laboratory of Public Health is the country's first medical laboratory with 50 000 to 70 000 medical laboratory tests realised per year. The present study set at the National Laboratory of Public Health in Gabon gives us a general trend on the CT and HIV evolution over the past years. It also gives a particular view on how population groups are affected by both CT and HIV infections. Although data from this study provides important information on STIs distribution and trends across gender and age groups, the LNSP prevalence rates cannot be generalized to the whole country as studies have shown differences between urban and rural populations [26], [27]. Also, the Gabonese setting is particularly interesting because in term of sexual behaviours, it provides us with an opportunity to study sexual lifestyles in a relatively educated African population.

Data showed 24% drop in the CT rate infection between 2007 and 2010, followed in 2011 by a 14% increase; while HIV infection rates for the same period remained relatively constant (around 15%–16%). Furthermore, the risk of a CT-positive subject getting HIV was not higher than the risk of a CT-negative subject getting HIV. This could be explained by the high prevalence of CT-positive subjects among the HIV-negative population and the prevalence of HIV-positive, which is higher than CT-negative subjects compared to CT-positive subjects. Although, the plateauing of the HIV rate observed over the past 5 years is good news, it should not be a cause of relaxation; because the increase of CT if not controlled portends a spike in HIV cases as the rise of CT indicates an increase in unsafe sexual practices.

Also the analysis of our data revealed no positive association between HIV and CT infections. The absence of association observed here could be explained by (1) Libreville low HIV prevalence (3.9%) [28] and by (2) the huge gap or difference in the recorded number of both infections. But these explanations may not be sufficient. The limitation of this section of the study is that we did not investigate whether HIV positive people changed their sexual behaviors to prevent themselves and others from getting infected with STIs.

Between 2007 and 2011, 65.2% of people who had STIs were young adults aged between 18 and 35 years old. Similar patterns are observed in the developed world [29], [30]. The high rate of sexual activity among young adults may explain why they represent more than half of people who had STIs.

Under-aged people (<18 years old) engaging in sexual activities should be a concern in emerging countries. Studies on adolescent sexual behavior in sub-Saharan Africa revealed the hazardous character of sexual encounter in young people, thereby predisposing them to the risks of STIs [31]. In our setting 4.2% of people with STIs and 3.2% of people who had HIV were under aged children (<18 years old). We observed between 2007 and 2011 a decline in the contribution of adolescents to the general epidemiology of STIs. This decline could be attributed to the multiplication in the recent years of STIs prevention campaigns targeting adolescent and school children. However with 22% of under aged girls having more than one sex partner compared to 11.6% of young adult women, under aged girls seemed to be more vulnerable.

Another matter of concern is the inconsistent use of condoms by our population (about 80% of women and 66% of men). As demonstrated by Wand et al., [32] inconsistent use of protection during sex is highly associated with STIs. We observed that inconsistent use of condoms was higher in men with lower level of education. The rate of men with multiple partners was 4 fold higher than the women rate. Sex with non-spousal or non-life partners reached 46.3% in men and 8.5% in women.

Male students and men who represented a higher socioeconomic level engaged themselves the most in sexual intercourse with multiple partners. Risky sexual behaviors among students are well documented [33]. Also documented is the positive association between risky sexual behaviors and wealth index [34], [35], which Bingenheimer JB., [36] explained by: men's control of financial resources and freedom from social control mechanisms embedded in society.

Sex with multiple partners was the highest in educated workingwomen and female students than other women (unemployed and employed with a lower level of education). Others have also reported similar findings in sub-Saharan Africa [5], [37]. This could be explained by the initial state of women acceding to emancipation, a state where they are bridging all sexual taboo and experimenting their sexuality as men do, a state of “irrational exuberance”. Also, In the United States of America, Annang et al., [38] showed that determinants such as education, although an important factor associated with STIs prevalence, it has a differential impact on ethnic groups. Our data also showed that sexual activity and the percentage of men reporting more sexual partners was higher than the one of women. A possible explanation is that that men are more sexually assertive than women. Also, some men gauge their virility in terms of how many sexual partners they have therefore, they may feel compelled to be truthful or even exaggerate. However, women, believing that they should have few partners, may minimize their past [39], [40].

Our study suggests that poverty is no longer the principal driver of STIs epidemic, as sexual risk-taking behaviors that put people at risk seem to increase with wealth in developing countries [34], [35]. Therefore, programs against STIs should design intervention strategies that take into consideration the specific psychology of society groups. Understanding the specific psychological state of society groups could lead to the understanding of why in the developing world or in a given ethnic group increased awareness is not always associated with a reduction in STIs.

We hope that our study will contribute towards improving and understanding STI epidemiology and in informing the development of future public health strategies in the field of sexual health.
