# Correction: *Chlamydia trachomatis*, Human Immunodeficiency Virus (HIV) Distribution and Sexual Behaviors across Gender and Age Group in an African Setting

**DOI:** 10.1371/journal.pone.0115365

**Published:** 2014-12-05

**Authors:** The PLOS ONE Staff

There are a number of errors in the legends for Table 2 and Table 3. The legends for Table 2 and Table 3 are incorrectly switched. The legend that appears with Table 2 should appear with Table 3, and the legend that appears with Table 3 should appear with Table 2. Additionally, the legend for Table 2 should not include the words “gender and”. Please view the correct Table 2 and Table 3 with legends here:

**Table 2:** Chlamydia trachomatis (CT) and the human immunodeficiency virus (VIH) infections distribution across age groups.

	2007		2008		2009		2010		2011	
	Chlamydia	HIV	Chlamydia	HIV	Chlamydia	HIV	Chlamydia	HIV	Chlamydia	HIV
**All subjects**	1657	12	2546	398	1248	285	1554	388	1809	323
**Under 18 years old**	101 (6.1%)	0 (0%)	138 (5.4%)	20 (5%)	113 (9%)	15 (5.3%)	35 (2%)	2 (0.5%)	23 (1.3%)	11 (3.4%)
**18-35 year old**	1164 (70.2%)	7 (58%)	1910 (75%)	238 (60%)	866 (69.5%)	172 (60.4%)	1025 (66%)	211 (54.4%)	1099 (60.7%)	149 (46.1%)
**Over 35 year old**	392 (23.7%)	5 (42%)	498 (19.6%)	140 (35%)	269 (21.5%)	98 (34.4%)	494 (32%)	175 (45.1%)	667 (37%)	163 (50.5%)
**Pupils or Students**	450 (27.1%)	0 (0%)	1224 (48%)	95 (24%)	404 (32.4%)	35 (12.3%)	504 (32.4%)	36 (9.3%)	391 (21.6%)	21 (6.5%)

**Table 3:** Chlamydia trachomatis (CT) and human immunodeficiency virus (VIH) infection prevalence for the period 2007–2011.

Patients tested for both infection between 2007 and 2011				
	HIV (+)	HIV (-)	Total	HIV-prevalence rate
**Chlamydia (+)**	83	911	994	8.3%
**Chlamydia (-)**	97	763	860	11.3%
**Total**	180	1674	1854	9.7%
**CT- prevalence**	46.1%	54.4%	53.6%	
